# Lecithin as an Effective Modifier of the Transport Properties of Variously Crosslinked Hydrogels

**DOI:** 10.3390/gels9050367

**Published:** 2023-04-27

**Authors:** Richard Heger, Natalia Zinkovska, Monika Trudicova, Martin Kadlec, Miloslav Pekar, Jiri Smilek

**Affiliations:** Institute of Physical and Applied Chemistry, Faculty of Chemistry, Brno University of Technology, 61200 Brno, Czech Republic; xczinkovska@fch.vut.cz (N.Z.); xctrudicova@fch.vut.cz (M.T.); martin.kadlec@vut.cz (M.K.)

**Keywords:** lecithin, hydrogel, diffusion, scanning electron microscopy, extracellular matrix, transport properties, model drugs

## Abstract

Transport properties are one of the most crucial assets of hydrogel samples, influencing their main application potential, i.e., as drug carriers. Depending on the type of drug or the application itself, it is very important to be able to control these transport properties in an appropriate manner. This study seeks to modify these properties by adding amphiphiles, specifically lecithin. Through its self-assembly, lecithin modifies the inner structure of the hydrogel, which affects its properties, especially the transport ones. In the proposed paper, these properties are studied mainly using various probes (organic dyes) to effectively simulate drugs in simple release diffusion experiments controlled by UV-Vis spectrophotometry. Scanning electron microscopy was used to help characterize the diffusion systems. The effects of lecithin and its concentrations, as well as the effects of variously charged model drugs, were discussed. Lecithin decreases the values of the diffusion coefficient independently of the dye used and the type of crosslinking. The ability to influence transport properties is better observed in xerogel samples. The results, complementing previously published conclusions, showed that lecithin can alter a hydrogel’s structure and therefore its transport properties.

## 1. Introduction

Hydrogels are hydrophilic polymeric materials with a three-dimensional structure (or 3D networks of hydrophilic polymers) capable of swelling in aqueous solutions or biological fluids and also possessing unique properties, such as biocompatibility, biodegradability, superabsorbency, viscoelasticity, softness, and responsiveness to external stimuli (pH and temperature) [1,2,3]. The presence of these properties makes hydrogels a promising material in biomedicine, tissue engineering, and especially the preparation of drug-delivery systems with controlled release [4,5].

In the case of designing systems for the controlled release and delivery of drugs, special attention should be given to the mechanism of transportation and its correlation with the microstructure of hydrogels. The physical and mechanical properties of such systems can easily be improved (1) by adding a structure modifier; (2) by means of binary systems composed of two or more mixed polymers, such as interpenetrating polymer networks; (3) by using self-reinforced composite materials composed of fibers embedded in a matrix of the same polymer; and (4) by means of the sol-gel reaction to produce silica reinforcement [6]. Another alternative way to enhance the mechanical properties of hydrogel materials is to incorporate nanomaterials, such as silica, graphene, graphene nanotubes, carbon nanotubes, and plant fiber-based nanofibers, etc. For example, Mallikarjun B. Jalageri and G. C. Mohan Kumar, in their article, described a new material for designing cartilage scaffolds based on polyvinyl alcohol/polyvinyl pyrrolidone hydrogel reinforced by hydroxyapatite [7]. The addition of hydroxyapatite nanorods (HNr) improves the mechanical properties and morphological characteristics of PVA/PVP hydrogels. Soher N. Jayash et al. prepared novel hybrid hydrogels based on chitosan and silica using the sol-gel technique [8]. Such hydrogel systems exhibited improved degradation and mechanical properties, no significant cytotoxic effects, and high loading efficiencies. Eric A. Appel and coauthors developed self-assembled hydrogels prepared by means of non-covalent interactions between hydroxypropylmethylcellulose derivatives (HPMC-x) and core-shell nanoparticles (NPs) [9]. This approach enabled the creation of materials with a dual loading of hydrophobic and hydrophilic molecules and high biocompatibility, which are appropriate for controlled drug delivery applications.

The characterization of hydrogels with a modified internal structure is often based on the analysis of their mechanical properties as well as their biocompatibility and biodegradability. In our previous paper [10], we provided a detailed and comprehensive analysis of the impact of lecithin (a naturally occurring mixture of phosphatides) on the morphological and rheological properties of hydrogels with different crosslinking mechanisms. The kinetics of drying and further swelling of these systems were studied as well. In addition to this valuable information, it is also essential to study the transport behavior of these materials in light of their potential application as controlled release systems.

Transport properties generally include viscosity, thermal conductivity, and the diffusion coefficient (diffusivity), which indicates the rate at which specific (per unit volume) momentum, heat, or mass are transferred [11]. Studying diffusion in hydrogels is extremely important in order to predict diffusant transport with respect to their potential application as drug delivery systems and also in order to extract information on polymer network structures [12]. Therefore, it should be kept in mind that the choice of an appropriate diffusion measurement method is essential since there are a huge number of different methods and instrumental techniques, as well as their combinations, and none of them is universal. Furthermore, experimental methods can be divided into a few main categories. The first (traditional) comprises concentration gradient methods (steady-state), in which a diffusional steady-state flux is set up through a gel diaphragm separating two liquid-filled, stirred compartments, and the effective diffusion coefficient is calculated. The second comprises concentration gradient methods (non-steady-state). This category includes, for example, uptake/release from a hydrogel immersed in a stirred solution [13]. In this case, the decrease or increase in the amount of diffusant in the outer solution is measured, and subsequently, the diffusion coefficient is calculated. The next category covers advanced instrumental techniques, such as diffusion-sensitive NMR, which enables the evaluation of solute self-diffusion [14,15,16]; fluorescence recovery after photobleaching (FRAP), which involves tracking the intensity distribution around the photobleached spot over time, as well as the use of powerful mathematical tools to characterize the diffusion coefficients of diffusants [17]; and fluorescence correlation spectroscopy (FCS), which allows the direct measurement of the translational diffusion of solutes [18,19]. The last category includes computer simulation studies on solute diffusion in gels, such as coarse-grained (CG) simulations, which are widely used to simulate polymer networks and also to predict the impact of formulation and processing parameters on the kinetics of drug release [20,21].

In this article, we present a study of the transport properties of hydrogel and xerogel systems determined on the basis of the given release experiments. In general, we investigated the diffusion of organic dyes (rhodamine 6G as a model of positively charged molecules; eosin B and amido black 10 B as models of negatively charged molecules) and calculated the diffusion coefficients for hydrogel systems with different weight concentrations of lecithin as a structure modifier.

## 2. Results and Discussion

According to previously published data and to extend the results obtained in the previous article [10], the same types of crosslinking and materials were chosen. For the formation of hydrogel matrices, the following materials were used: the linear polysaccharide agarose, as an example of physical crosslinking; sodium alginate crosslinked by calcium chloride, as an example of an ionic type of crosslinking; and poly(vinyl alcohol)-chitosan crosslinked by epichlorohydrin, as an example of a hydrogel with chemical crosslinking. Crosslinking is one of the main factors that influences the transport properties of the hydrogel (Figure S1), and thus it is important to take this factor into account when trying to characterize the influence of lecithin on the different hydrogel and xerogel samples.

For each type of crosslinking, samples differing in lecithin concentration were investigated. Lecithin concentrations were selected according to a previous study, and the samples were labeled according to these concentrations (“0.5”, “1”, and “2”); the reference sample without lecithin addition was marked as “R”.

The probes that were chosen to study the transport properties of the hydrogel samples differed in some of their physicochemical properties (electric charge and molecular weight) as well as in some of their visual properties, such as their absorption maximum. Specifically, the probes chosen were the positively charged rhodamine 6G (479.02 g·mol^−1^) and the negatively-charged dyes eosin B (580.09 g·mol^−1^) and amido black 10B (616.50 g·mol^−1^).

Crucial transport parameters were obtained for hydrogel and xerogel samples placed in the container filled with distilled water, and the amount of released dye was controlled and measured by a UV-Vis spectrophotometer set up for hydrogels. In the case of xerogels, the same optical probes were used, but due to the very fast release of dye from the xerogel samples, more frequent measurements were recorded (also using UV-Vis). The supporting method was scanning electron microscopy (SEM), from which images of the studied hydrogels were obtained and further compared with previously published xerogel images.

### 2.1. Physical Crosslinking

To characterize the transport properties of the physically crosslinked hydrogel matrix, agarose hydrogels modified by different additions of lecithin were studied. Agarose was chosen because, in the context of amphiphilic modifications, we try to approach the functionality and properties of extracellular matrix (ECM), and agarose hydrogels are frequently used in vitro models across numerous disciplines [22]. Hydrogel samples, as well as dried xerogel samples, were prepared with a chosen dye already incorporated into the structure (the original hydrogel samples were prepared using a solution of the dye). A schematic figure of the preparation procedure can be seen in the Supplementary materials, Figure S2.

#### 2.1.1. Dye-Release Experiments

The effective diffusion coefficient (*D*_eff_) was the crucial transport parameter that provided us with information about the transport properties of the studied hydrogels. Specifically, it is a value that describes how each individual dye is released from the hydrogel. The procedure for obtaining *D*_eff_ and the method to calculate it are described in Section 4.2.

**Figure 1:**
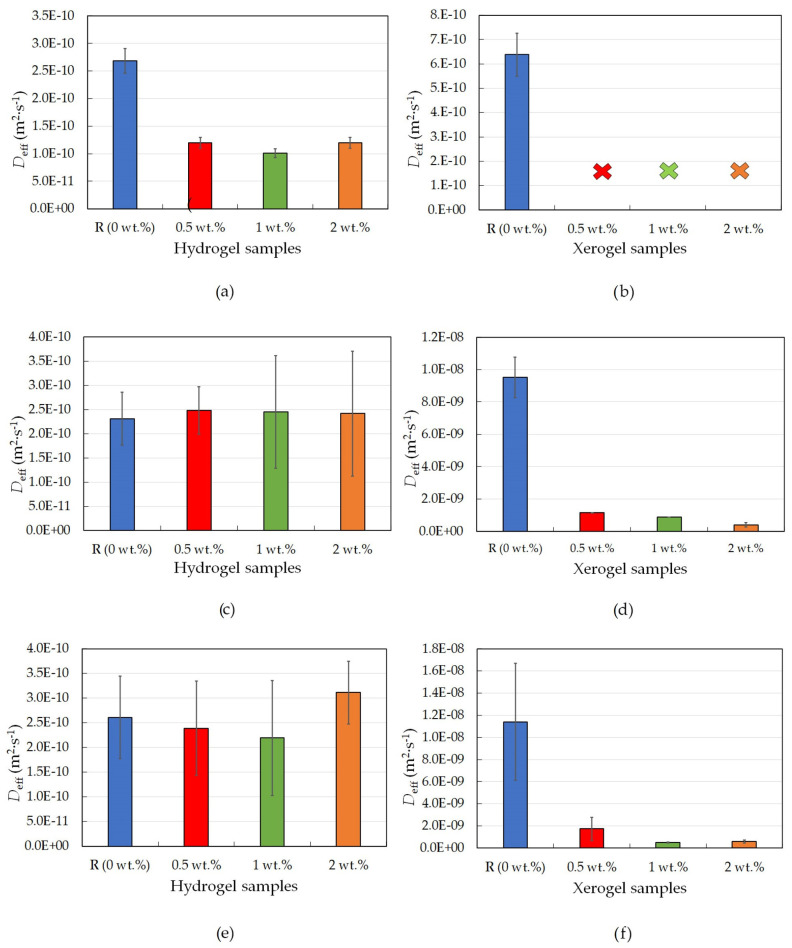
Effective diffusion coefficients for physically crosslinked agarose hydrogel and xerogel samples with different lecithin concentrations (0, 0.5, 1, and 2 wt.%); rhodamine 6G hydrogels (**a**), rhodamine 6G xerogels (**b**); eosin B hydrogels (**c**); eosin B xerogels (**d**); amido black 10B hydrogels (**e**); and amido black 10B xerogels (**f**).

Rhodamine 6G was chosen as the only positively charged probe due to its suitable molecular weight (comparable with that of cationic drugs, e.g., loperamide or ebastine) and previous experience with this organic dye [23]. The results obtained for agarose hydrogel samples modified by lecithin suggest that lecithin addition influences the transport properties of agarose hydrogels by decreasing the value of the diffusion coefficient (as seen in Table 1 and Figure 1a), which means that, in our study, the release of the model organic probe was significantly slowed down. The large deviations between individual measurements could have been caused by the use of different probes or their calibration. However, in each set of measurements, a trend of decreasing diffusion coefficient with increasing lecithin concentration was evident. The reference sample immediately released more dye than all the other samples, while the samples with lecithin released the positively charged dye significantly more slowly. This could be the result of the modified internal structure of these hydrogels due to the self-assembly of lecithin inside the hydrogel. The presence of lecithin, as well as interactions between the positively charged rhodamine 6G and the negatively charged functional groups of the lecithin molecule, enable the dye inside the hydrogel structure to become partially blocked and thus slow down or delay its release. This would also be confirmed by a number of publications dealing with obstructions inside agarose hydrogels [24,25].

What happens to the hydrogel after evaporation of the dispersion medium, and how does this affect the other substances in the gel? To answer this key question, the same hydrogel samples as used in the previous experiment were dried prior to characterization. In this case, however, the dye release from agarose was very quick, and it was not possible to measure the release over longer intervals as was performed for the dye release from non-dried hydrogel samples. For this reason, a fiber UV-Vis spectrometer was used, allowing the measurement of UV-Vis spectra every twenty seconds. In this setup, the absorbance was measured for approximately the first hour, which, for agarose xerogel samples, was enough time for around twenty percent of the dye to be released from the sample (for the reference sample, see Figure 1b). From the measured absorbance, the concentration was calculated.

**Figure 2:**
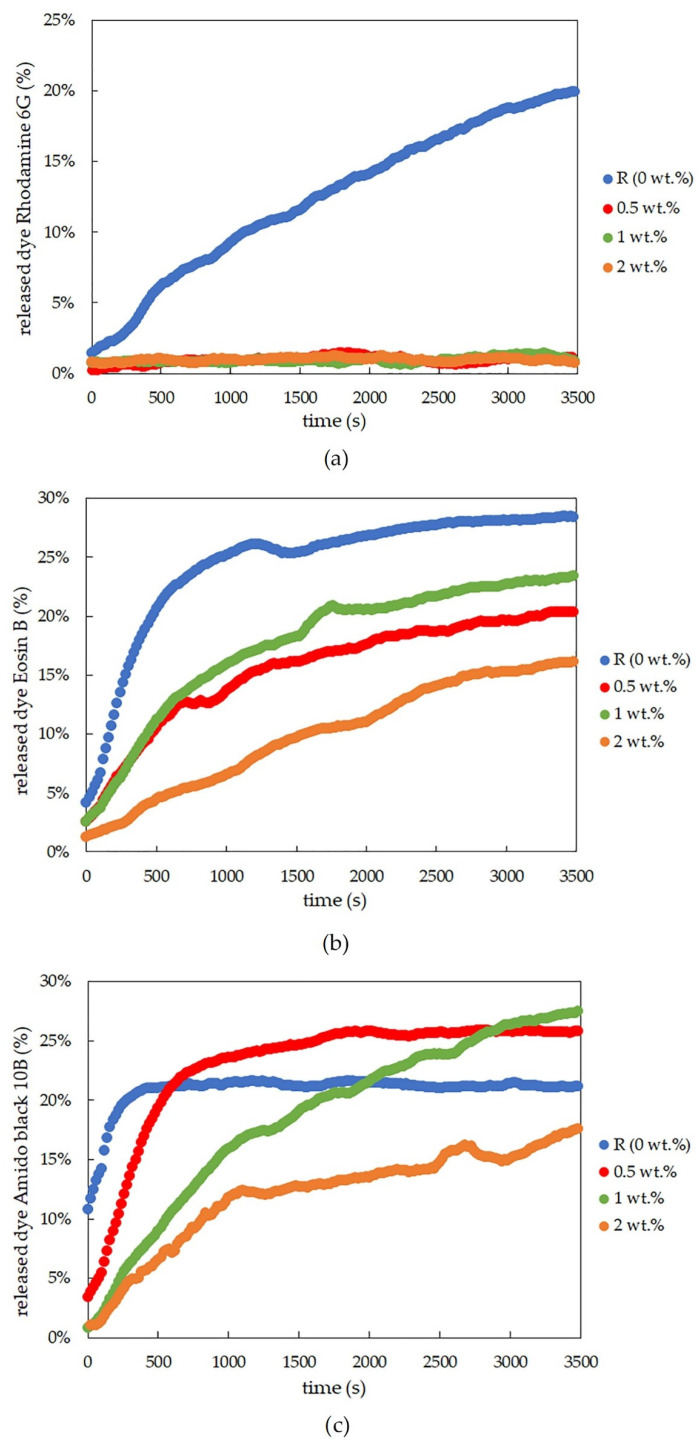
Time development of dye-release experiments for rhodamine 6G (**a**), eosin B (**b**), and amido black 10B (**c**), controlled by the optical probes for physically crosslinked agarose xerogels with different concentrations of lecithin.

The results from the dye-release experiments obtained for agarose xerogels loaded with rhodamine 6G confirmed the previously mentioned conclusions in Section 2.1.1. Therefore, the rhodamine 6G interacted with the lecithin modifier. This was particularly evident from the fact that it was not possible to obtain adequate values for the effective diffusion coefficient after the addition of lecithin. This hypothesis is also supported by the graphical representation of the release of the dye from the xerogel system (Figure 2a). These findings from Figure 1b show that the diffusion coefficient for the reference sample without lecithin was the only value obtainable for xerogels from the diffusion coefficient calculations (Table 1). For the lecithin additions, the amount of released dye, and thus the diffusion coefficient, was not measurable, and with increasing time, deformation of the samples occurred, which would again support the assumption of interactions between lecithin, rhodamine 6G, and agarose.

Other drug models used were negatively charged eosin B and amido black 10B. These were chosen to study oppositely charged models and to avoid interactions with the modifier. The second studied model dye was the negatively charged eosin B. The characterization of the dye-release process in physically crosslinked hydrogels and xerogels tuned by the addition of lecithin was analogous to rhodamine 6G measurements and has already been described in Section 2.1.1. At the start of the experiment, the dye-release process was similar for all hydrogel samples. With the passage of time, the release of dye accelerated (leading to a higher concentration of dye outside of the sample) in the reference sample and in samples with smaller lecithin concentrations (Table 2). The associated diffusion coefficients were ultimately almost identical for all samples, which is explained by the fact that eosin B and hydrogel matrix interactions with each other should not occur (on the basis that eosin B can react with positively charged components [26]), and thus the diffusion of the negatively charged eosin B is not slowed down inside the hydrogel. The results of FTIR analysis using PCA showed that the main differences between the agarose gels without lecithin and gels with lecithin spectrally correspond to the characteristic bands of lecithin (Figures S3 and S4).

The results for physically crosslinked agarose xerogels and eosin B are presented in Table 2 and Figure 1d. The diffusion coefficient is greater for the reference sample than for samples with lecithin additions (0.5 and 1 wt.%), with the coefficient being the lowest for the sample with the highest lecithin concentration (2 wt.%). This suggests that lecithin heavily influences the coefficient and that even small additions decrease the coefficient significantly. The greater the concentration of lecithin, the lower the *D*_eff_ value, while the differences between the *D*_eff_ values themselves were not so substantial. However, if we ignore the reference sample, the magnitude of the diffusion coefficient decreases linearly with increasing lecithin concentration. It can be predicted that the value would decrease even lower to the point where the addition of the modifier was too great, and the dye release was almost negligible or even ceased altogether. This hypothesis is also supported by the time development of the release of eosin B (Figure 2b), which shows the fastest initial release in the reference sample. For samples with different concentrations of lecithin, it is clear that the lecithin inside the system caused a gradual slowing of the release. Samples with smaller lecithin additions (0.5 and 1 wt.%) were almost indistinguishable from each other, and the time of release was very similar. The highest lecithin addition (2 wt.%) led to the biggest retardation.

The third and last studied model dye was negatively charged amido black 10B, and the corresponding results for hydrogels are summarized in Table 3 and Figure 1e. These exhibit the same trend as the results obtained for the other negatively charged dye, eosin B (Table 2 and Figure 1c); thus, the kinetics of the released dye are very similar to each other, which is also confirmed by the calculation of very similar diffusion coefficients. Thus, it can be said that for a negatively charged model drug, the lecithin within the hydrogel does not heavily influence its release time, and the drug is easily released.

The conclusions for amido black 10B within physically-crosslinked agarose xerogels are similar to those for eosin B (Table 3 and Figure 1f). The diffusion coefficient decreases with increasing lecithin concentration in hydrogel samples in comparison with the reference sample, in which no additional factors influence the transport of amido black 10B dye. As seen from Table 3, the higher the concentration of lecithin, the lower the value of the diffusion coefficient obtained. In the case of the highest lecithin concentration (2 wt.%), dye release was substantially slowed down, probably due to the specific lecithin structures formed inside the hydrogel structure. This is also supported by the timing of the release (Figure 2c). These results are very similar to those obtained for eosin B, with the only difference being the smallest addition of lecithin (0.5 wt.%), which is more similar to the reference sample.

#### 2.1.2. Morphological Characterization

The morphological characterizations of physically crosslinked agarose hydrogels complement the already-published SEM images of xerogel samples used in this study (SEM images already published in [10]). As stated in Section 4, hydrogel samples were shock-frozen in liquid nitrogen and immediately underwent lyophilization. Subsequently, the hydrogel structures were analyzed by SEM. The shock-freezing of samples was used to minimize the alteration of hydrogel structure caused by ice crystal growth, which can create some undesirable artifacts. Thus, the structures of freeze-dried hydrogels were similar to their structures in their native state [27].

**Figure 3:**
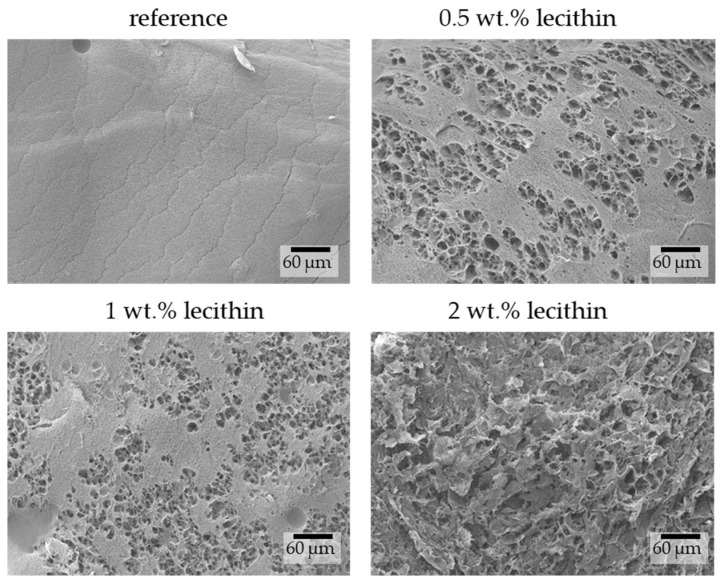
Surface morphologies of physically crosslinked agarose hydrogels with the addition of lecithin revealed by SEM. Original magnification: 500×.

**Figure 4:**
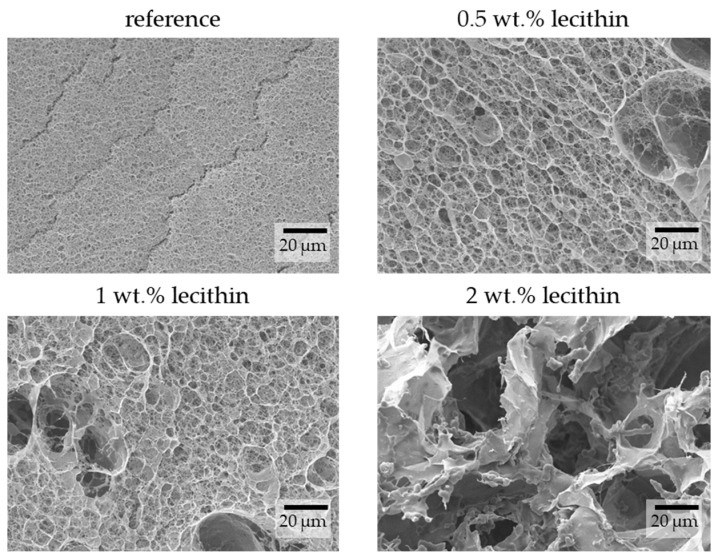
Surface morphologies of physically crosslinked agarose hydrogels with the addition of lecithin revealed by SEM. Original magnification: 5000×.

The images obtained from SEM show differences in hydrogel structures between samples with different lecithin concentrations (Figure 3) and how these structures are potentially affected by the lecithin addition. With increasing concentrations of lecithin, the structures of hydrogels seem more altered and collapsed. Lecithin, either lecithin alone or some structure formed from it, was probably trapped in the pores of the hydrogel network, causing them to enlarge or making them more sensitive to changes caused by the growth of ice crystals. This corresponds with results from the dye-release experiments, where lecithin slowed down diffusion in hydrogels due to specific interactions between them or simply by its presence in a particular place (i.e., “obstruction effect”). At a magnification of 5000× (Figure 4), it is possible to observe pores in the structure of hydrogels (except in the sample with the highest concentration of lecithin, where the structure collapsed). These pores correspond in size to the pores in the structure of agarose hydrogels found in previous work [27].

### 2.2. Ionic Crosslinking

As an example of an ionically crosslinked hydrogel matrix, sodium alginate crosslinked by calcium ions (2:1 wt. ratio) was studied. Bond formation resulted from the interaction of negatively charged poly(guluronic) acid units of alginate with divalent Ca^2+^ ions. Alginate has been extensively used as a hydrogel-synthetic ECM and thus is an ideal representative of an ionically crosslinked hydrogel for this study [28]. Structural modifications were evoked by lecithin additions, and the resulting transport properties were observed and compared by means of diffusion dye probes. A schematic figure of the preparation procedure can be seen in Supplementary Materials, Figure S5.

#### 2.2.1. Dye-Release Experiments

**Figure 5:**
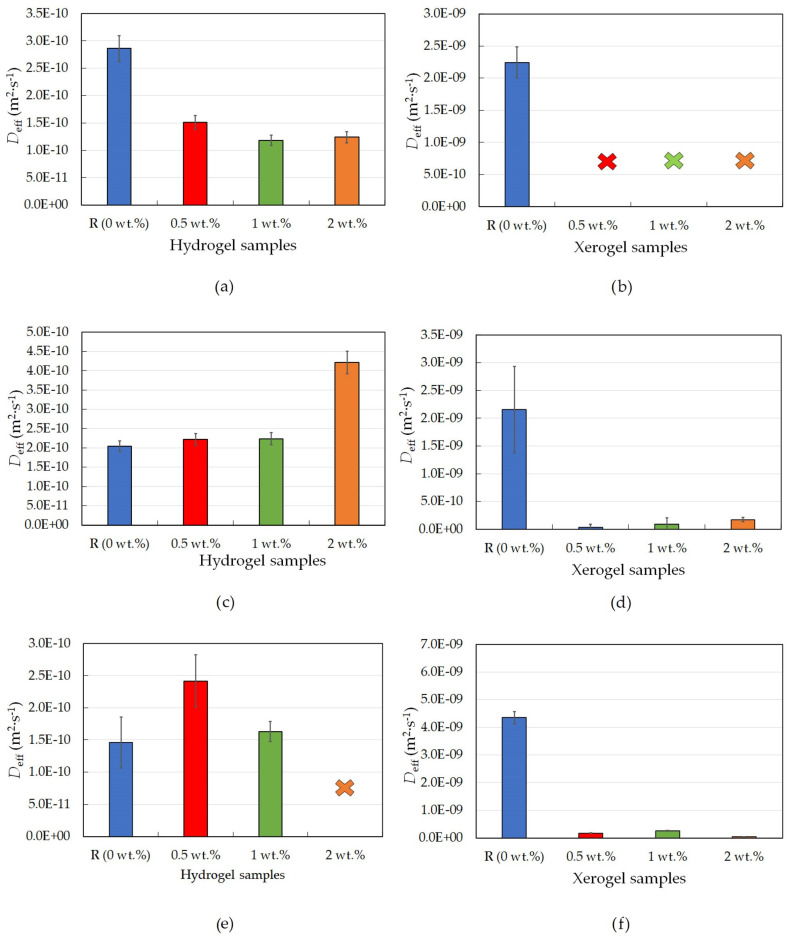
Effective diffusion coefficients for ionically-crosslinked alginate hydrogel and xerogel samples with different lecithin concentrations (0, 0.5, 1, and 2 wt.%); rhodamine 6G hydrogels (**a**), rhodamine 6G xerogels (**b**); eosin B hydrogels (**c**); eosin B xerogels (**d**); amido black 10B hydrogels (**e**); and amido black 10B xerogels (**f**).

The first studied model dye was positively charged rhodamine 6G, which was released from an ionically crosslinked alginate hydrogel. Lecithin, which acts as an internal structure modifier, was added to the hydrogel systems in different concentrations. The amount was the key factor that influenced both the hydrogel and xerogel structures and their related transport properties, which can be seen in Table 4 and Figure 5a.

The amount of released dye was the highest for the reference sample, as this hydrogel did not contain any structures or artifacts that would serve as obstacles or as storage moieties for the dye, and since the alginate hydrogel complex should act as a neutral system [29], the dye could be released out of the hydrogel without any problems. The kinetics of dye release slowed down with increasing lecithin concentration, i.e., with an increasing number of lecithin structures acting as obstacles. The associated effective diffusion coefficient (*D*_eff_) was the highest for the reference sample and decreased with increasing lecithin concentration. The differences in *D*_eff_ values for the samples with distinct additions of lecithin were negligible and thus confirmed the insignificant influence of lecithin concentration on the final value of the diffusion coefficient (in comparison with the reference sample).

**Figure 6:**
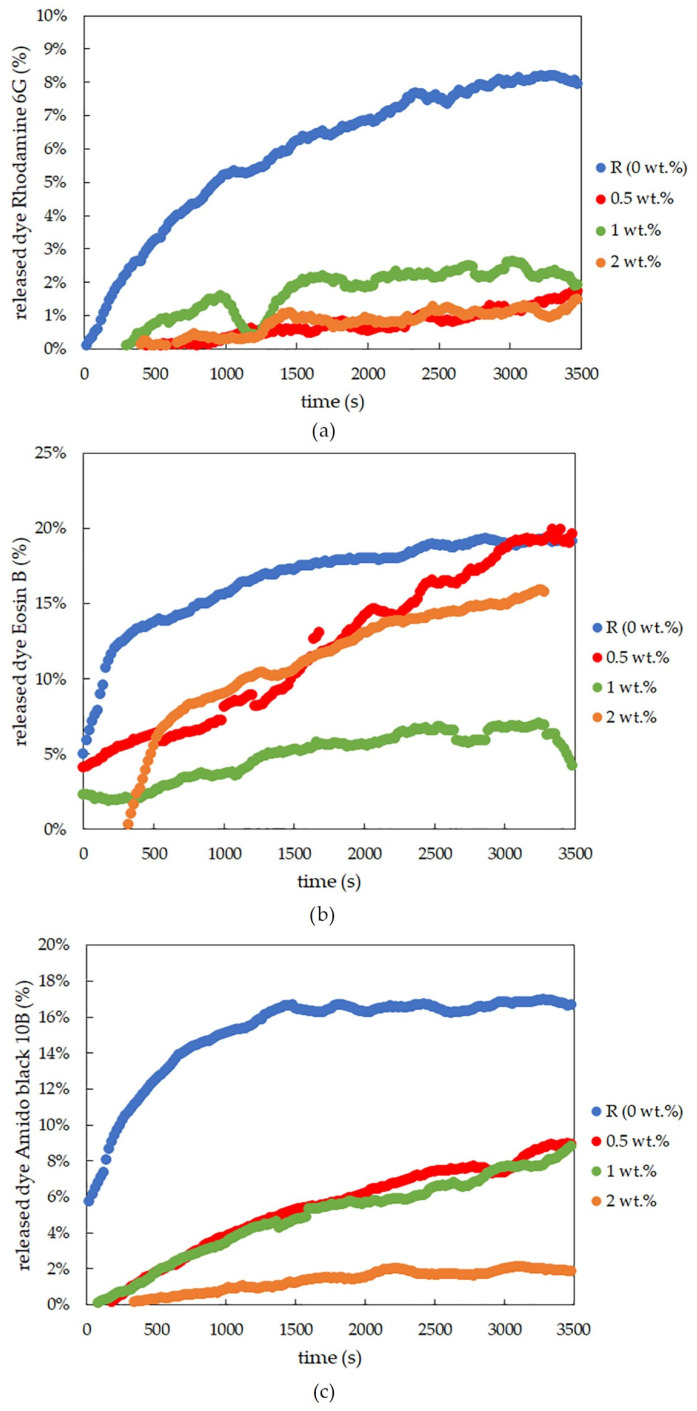
Time development of dye release for rhodamine 6G (**a**), eosin B (**b**), and amido black 10B (**c**), determined by optical probes for ionically crosslinked alginate xerogels with different concentrations of lecithin.

Xerogel samples for ionically crosslinked alginate yielded very similar results to those for physically crosslinked agarose samples. In the case of lecithin additions, the concentration of dye was undetectable, probably because of the blockage of positively charged dye release caused by lecithin throughout the whole duration of the experiment. The time of development of the dye release (Figure 6a) was almost identical to that for physically crosslinked hydrogels. The positively charged dye was almost immeasurable outside of the hydrogel, thus confirming interactions with the dye as well as the blockage caused by the lecithin additions [30].

Negatively charged dyes were also studied. As is obvious from Table 5, the increasing lecithin concentration slowed down the release of the negatively charged dye eosin B from ionically crosslinked alginate hydrogels. The diffusion coefficient was then very similar for each sample, regardless of the amount of lecithin within each hydrogel. The sample with the highest lecithin concentration (2 wt.%) presented high deviation values, which were caused by the high content of lecithin released from the sample with the dye.

The results for ionically crosslinked xerogels with negatively charged eosin B followed those for physically crosslinked xerogels with the same dye, showing that the reference sample had a much bigger diffusion coefficient than the other samples, their lower coefficients resulting from lecithin addition. According to this, it could be concluded that crosslinking does not have a considerable influence on the diffusion coefficients of xerogels for this type of model drug (dye). The timing of the release of this dye indicates that lecithin slowed down the release since alginate does not interact with eosin B [31]. As mentioned in the previous paragraph, the sample with the highest lecithin concentration (2 wt.%) initially had the slowest release, but, with time, it was possible to observe (Figure 6b) its acceleration, which was caused by the release of lecithin.

The release of the second negatively charged dye, amido black 10B, from ionically crosslinked alginate was significantly slowed down by lecithin additions. The associated diffusion coefficient increased with increasing concentration up to the 1 wt.% sample. During the experiment, the dye release from the samples with the two highest lecithin concentrations was either immeasurable or exhibited big deviations. This was caused by disintegration, which led to the release of both the dye and the lecithin. A similar order of magnitude for the diffusion coefficients (Figure 5c and Table 5) can be observed for the other negatively charged dye, eosin B; however, the differences between each sample for amido black 10b are obvious, which cannot be said for the eosin B samples. In comparison with physically crosslinked agarose hydrogels, again for amido black 10B (Figure 5e and Table 6), the coefficient has values in the same order of magnitude.

On the other hand, ionically crosslinked xerogels with negatively charged amido black 10B show the exact same trend as physically crosslinked agarose hydrogels. The value of the diffusion coefficient decreases with increasing lecithin concentration. The results of FTIR analysis using PCA showed that the main differences between the alginate gels without and with lecithin spectrally correspond to the characteristic bands of lecithin (Figures S3 and S6).

#### 2.2.2. Morphological Characterization

**Figure 7:**
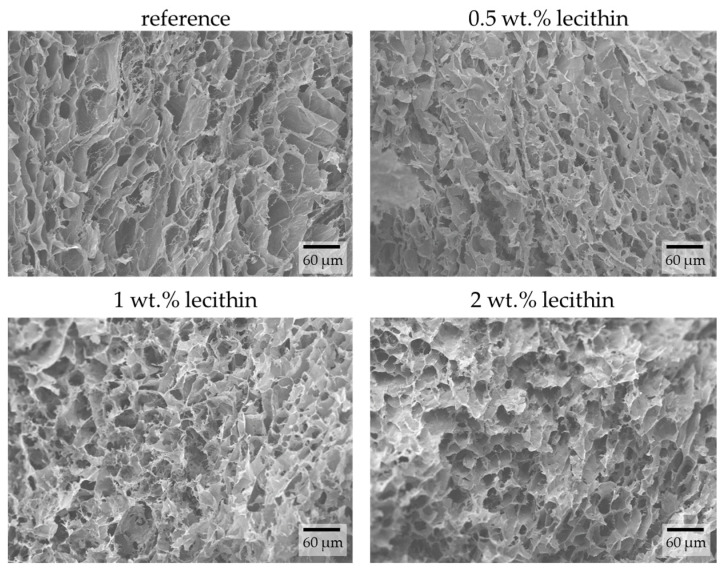
Surface morphologies of ionically crosslinked alginate hydrogels with the addition of lecithin revealed by SEM. Original magnification: 500×.

Prior to SEM observation, ionically crosslinked alginate hydrogels were shock-frozen in liquid nitrogen and subsequently lyophilized, as were the agarose hydrogels. As stated before, this procedure was used to minimize the alteration of hydrogel structure; however, for these types of hydrogels, it was not effective. As can be seen from Figure 7, the structure of all prepared hydrogels appears similar, leading to the conclusion that they are altered. This was most likely a result of the preparation of the samples. Since these hydrogels were prepared in a smaller volume than the agarose hydrogels, they may have been partially unfrozen prior to lyophilization, which would have caused ice crystal growth and thus the presence of structural artifacts [32]. These results therefore represent artifact-biased images of hydrogels in their native form.

### 2.3. Chemical Crosslinking

A mixture of PVA (poly(vinyl alcohol)) with chitosan crosslinked by epichlorohydrin was studied as an example of a chemically crosslinked hydrogel. As well as other types of crosslinking, the internal structure and subsequent transport properties of samples were modified by the addition of different amounts of amphiphilic lecithin. Chemically crosslinked samples were studied only in their xerogel form since the preparation method finishes with mixtures in their dried state; thus, rehydrated samples would yield the same results (Section 4.1). The only difference was in the morphological characterization, in which samples were rehydrated to observe their structures in hydrogel form. The samples were chosen for their close similarity to ECM and their ideal biomedical properties [33]. A schematic figure of the preparation procedure can be seen in the Supplementary Materials, Figure S7.

#### 2.3.1. Dye-Release Experiments

The main evaluated parameters were the diffusion coefficients and the amount of released dye. Three different dyes were used to study the transport properties of chemically crosslinked PVA-chitosan samples: positively charged rhodamine 6G, negatively charged eosin B, and amido black 10B. The negatively charged dyes were held inside the gel very strongly, which was because of the small pores in the highly organized structure of the chemically crosslinked gel as well as the interaction of the positively charged chitosan with the negatively charged dye. The only dye that was measurable outside the gel was released immediately after the experiment started; neither long nor short experiments, the latter focused on the first hour of the experiment, were able to provide results that could be analyzed. Only a residual amount of both of these dyes was released from the xerogels; therefore, no diffusion coefficient values could be calculated.

**Figure 8:**
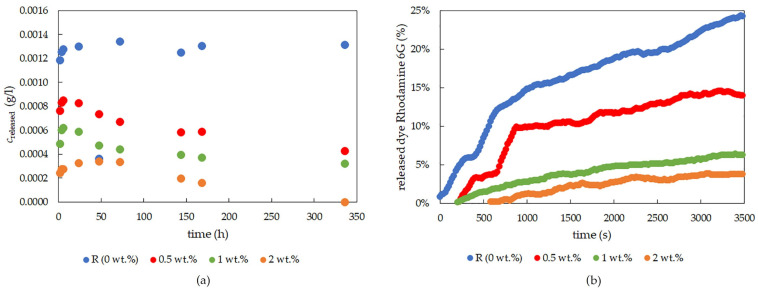
The concentration of rhodamine 6G released from xerogel samples over a longer period of time (**a**) and the time progress of dye-release experiments using rhodamine 6G for the first 60 min, determined by the optical probe for chemically cross-linked PVA-chitosan (**b**).

**Figure 9:**
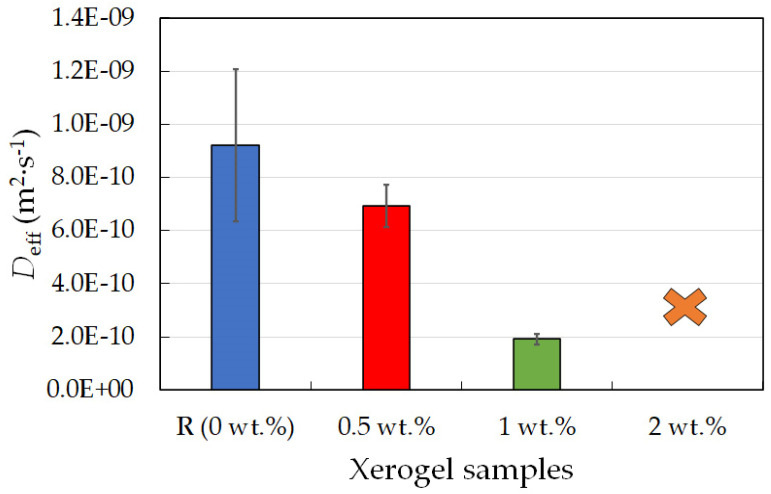
Effective diffusion coefficients for rhodamine 6G for chemically crosslinked PVA-chitosan xerogel samples with different lecithin concentrations (0, 0.5, 1, and 2 wt.%).

Only experiments with the positively charged dye, rhodamine 6G, yielded results sufficient for analyses and for obtaining information about diffusion coefficients. As can be seen in Figure 8a, the release of the dye was very fast, and the longer experiment setting was not appropriate for this type of gel. This burst release was studied on similar materials with the purpose of slowing the release [34]. Only experiments focused on the first hour of diffusion were able to yield results. Lecithin influenced the amount of dye released (Figure 8b), with the amount of released dye decreasing with increasing lecithin concentration. The same can be said about the diffusion coefficient (Figure 9 and Table 7), where the results obtained for the highest lecithin concentration (2 wt.%) are unequivocal since this lecithin concentration led to damage to the PVA-chitosan xerogel. The results of FTIR analysis using PCA showed that the main differences between the PVA-chitosan gels with and without spectroscopy correspond to the characteristic bands of lecithin (Figures S3 and S8).

#### 2.3.2. Morphological Characterization

**Figure 10:**
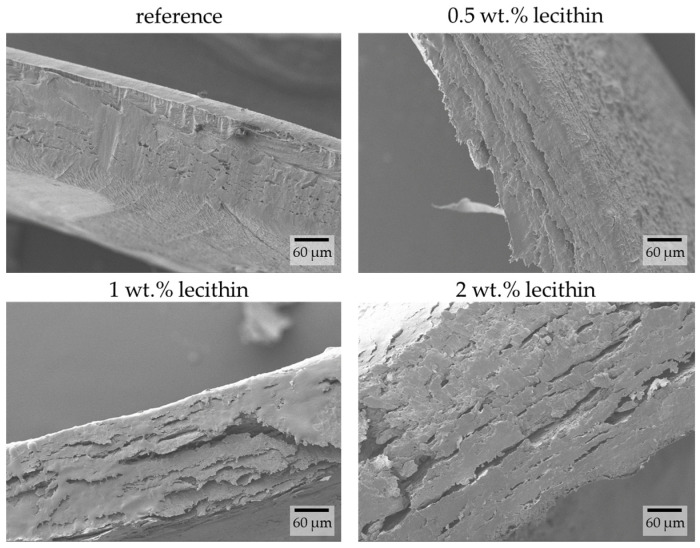
Cross-sectional view of chemically crosslinked PVA-chitosan hydrogels with the addition of lecithin revealed by SEM. Original magnification: 500×.

The chemically crosslinked hydrogels were used in their native (hydrated) form for the preparation of samples for morphological study. The prepared xerogels were left to swell in water and were subsequently shock-frozen in liquid nitrogen and freeze-dried. The images obtained using SEM show the layered structure of the hydrogels. The only observable difference between each sample is the smoothness of the cross-sectional view. With increasing lecithin content, the interlayer roughness increases (Figure 10).

These results correspond with already-published data for xerogels of these chemically crosslinked hydrogels [10]. Since the structures of xerogels and hydrogels for this type of material are very similar, it does not matter which preparation procedure is used to observe the structure using SEM. Furthermore, since the hydrogel structure is smooth and no pores are visible, these observations correspond with results for dye release, in which only a small amount of dye is released.

## 3. Conclusions

This work focused on the description of the transport properties of differently crosslinked hydrogels and xerogels (physically crosslinked agarose, ionically crosslinked alginate, and chemically crosslinked PVA-chitosan), which were altered with different concentrations of amphiphilic lecithin as a structure modifier to influence the transport properties of the gels. For characterization of the transport properties of the above-mentioned hydrogel systems, various model probes with different charges were used (positively charged rhodamine 6G, negatively charged eosin B, and amido black 10B).

The positively charged dye interacted with the lecithin modifier, which meant that for the physically and ionically crosslinked hydrogels, the dye was held within the hydrogel and released very little dye or none at all, regardless of the quantity of the modifier. When the charge of the dyes did not interact with the modifier (in the case of negatively charged dyes), the quantity of lecithin had a significant impact on the speed and amount of dye release. Which meant that the diffusion coefficient was lower for the xerogel samples with lecithin compared to the reference samples. In summary, therefore, we can state that the addition of lecithin to physically or ionically crosslinked hydrogels slows down the release of the model drug and affects the amount of drug released. Chemically crosslinked hydrogels appear to be a system that is also affected by lecithin, but not as significantly as in other types of crosslinking.

This work closely follows already-published results dealing mainly with the mechanical properties of the same types of hydrogels and modifications used in this work. Together with our former work, it is possible to compare different morphological characterizations of gels in their hydrogel and xerogel forms.

## 4. Materials and Methods

Differently crosslinked hydrogel matrices and the effect of lecithin on their properties were studied. Three different crosslinking mechanisms were studied: physical, ionic, and chemical. Agarose E was purchased from Condalab (Madrid, Spain), calcium chloride from Lach-Ner (Neratovice, Czech Republic), and sodium alginate, poly(vinyl alcohol), chitosan, epichlorohydrin, and L-α-Phosphatidylcholine (lecithin) were all purchased from Sigma-Aldrich (Prague, Czech Republic). Dyes used for diffusion experiments differed in charge: positively charged rhodamine 6G (Sigma-Aldrich, Prague, Czech Republic), negatively charged amido black 10B (Merck, Darmstadt, Germany), and eosin B (Sigma-Aldrich, Prague, Czech Republic) were used.

### 4.1. Sample Preparations

Physically crosslinked hydrogels were prepared using agarose. For every sample, a mixture of agarose (1 wt.%) and lecithin (0, 0.5, 1, or 2 wt.%) was weighed in a container. A liquid dispersion medium was then added to the mixture. The dispersion medium was either pure deionized water (ELGA, PURELAB Classic) or, if needed, a solution of appropriate dye (rhodamine 6G—0.01 g·l^−1^, eosin B—0.015 g·l^−1^, or amido black 10B—0.01 g·l^−1^). The sample thus prepared was mixed and dissolved at an elevated temperature. It was then left to stand for 24 h to gel prior to further study [10,23].

Ionically-crosslinked hydrogels were prepared by sodium alginate (2 wt.%) crosslinked by calcium ions of calcium chloride (0.1 mol·dm^3^) at a two-to-one weight ratio. Lecithin was added to the alginate solution and stirred for 24 h. A solution of calcium chloride (if needed, the calcium chloride was dissolved in a solution of an appropriate dye) was sprayed on the layer of sodium alginate with lecithin in order to create a homogeneous hydrogel [10,28]. The sample was then left to stand for 24 h in order for bond creation to finish and for the sample to relax. The hydrogels were then ready for further investigation.

For the preparation of chemically crosslinked hydrogels, lecithin was added to the PVA solution and stirred for 24 h [10], after which the procedure closely followed that described by Garnica-Palafox et al. [33].

### 4.2. Diffusion Release Experiments

The most important information concerning prospective drug carriers, which were mimicked by hydrogel samples in this study, is their ability to release the drug at a optimal speed. The diffusion/dye-release experiments were the core technique for this study. Samples with different crosslinking mechanisms were studied, as well as lecithin’s ability to influence this characteristic. Release characteristics were obtained for samples in their hydrogel form as well as for samples that were dried into their xerogel form. As model drugs released from the matrices, positively-charged rhodamine 6G, negatively charged eosin B, and amido black 10B were used.

The dye was added at the beginning of sample preparation. Thus, samples were prepared with the dye already in them. If the sample was supposed to be in xerogel form, hydrogels were dried to a constant weight in the laboratory oven at 45 °C. Hydrogel and xerogel samples were cut into the same-sized pieces and put into flasks with a defined volume of deionized water (hydrogels with 20 mL and xerogels with 40 mL) which was constantly stirred. The water was then routinely measured in a UV/Vis spectrophotometer every ten minutes to obtain the absorbance value for the released dye. The experiment ended when all the dye was released, the amount led to a constant weight, or the sample was damaged or destroyed.

The most fundamental parameter that can express (and consequently be used to compare) the transport properties of hydrogels with inner structural changes resulting from the addition of a modifier, such as lecithin, is the effective diffusion coefficient, expressed as the amount of dye released from the gel per m^2^∙s^−1^.

It must be noted that calculated diffusion coefficients are not absolute values but only relative (or effective) ones. This means that porosity and tortuosity are hidden in the values of diffusion coefficients. The determination of absolute values of diffusion coefficients requires deeper research focused specifically on the diffusion phenomena of each specific hydrogel. This was not the principal aim of this publication. Therefore, we used effective values for the diffusion coefficients as proxies for comparing the transport properties of the studied hydrogels as a function of the additive concentration and/or type of crosslinking.

The time of development of the mass of released dye can be expressed as follows:

(1) $$\frac{n_{t}}{n_{\mathrm{rov}}}=\frac{4}{\delta }\cdot \sqrt{\left(\frac{D_{\mathrm{gel}}}{\pi }\right)}\cdot \sqrt{t}$$

(2) $$k=\frac{4}{\delta }\cdot \sqrt{\left(\frac{D_{\mathrm{gel}}}{\pi }\right)}$$

(3) $$D_{\mathrm{gel}}={\left(k\cdot \frac{4}{\delta }\right)}^{2}\cdot \pi$$

The total amount of released dye at a specific time is expressed as the ratio of the amount of substance at a given time (*n*_t_) divided by the amount of substance at equilibrium (*n*_rov_). The effective diffusion coefficients were calculated from the slope of the linear regression of this ratio of the amount of substance as a function of the square root of time. The dimensions of the releasing medium (i.e., hydrogels) were considered as circles of diameter (δ). From this simple calculation, it is obvious that the release experiments must be performed until the equilibrium state is achieved (when the concentration of released dye does not change over time) [35].

During the measurements as well as after evaluation of the results from UV-Vis spectrophotometry, it was found that the release of the dyes from the xerogel samples was too fast for the experimental setup. For this reason, the xerogels were measured again using optical probes and OceanView 2.0 software (OceanInsight, Orlando, FL, USA), which allowed us to obtain UV-Vis absorbance every 20 s for the first hour of the experiment.

### 4.3. Scanning Electron Microscopy

Morphological characterization of the structures of all prepared hydrogel samples was performed using a ZEISS EVO LS 10 (Carl Zeiss AG, Germany) scanning electron microscope. The samples in their native (i.e., water-filled) form were shock-frozen in liquid nitrogen and then immediately freeze-dried. A few small specimens were taken from each studied hydrogel to maintain objective observation. Before observation, the specimens were gold-coated in a sputtering device (POLARON, Quorum Technologies, Lewes, UK).

The surface morphologies of every sample were recorded. Observations were realized in secondary electron mode, and the accelerating voltage was set to 5 kV to avoid charging the sample.

## Figures and Tables

**Table 1:** Effective diffusion coefficients calculated from dye-release experiments for physically crosslinked agarose hydrogels and xerogels with different concentrations of lecithin and the positively charged dye rhodamine 6G.

Rhodamine 6G	Hydrogel *D*_eff_ × 10^−10^ (m^2^·s^−1^)	Xerogel *D*_eff_ × 10^−10^ (m^2^·s^−1^)
R (0 wt.%)	2.7 ± 0.2	6.4 ± 0.9
0.5 wt.%	1.2 ± 0.1	—
1 wt.%	1.0 ± 0.1	—
2 wt.%	1.2 ± 0.1	—

**Table 2:** Effective diffusion coefficients calculated from dye-release experiments for physically crosslinked agarose hydrogels and xerogels with different concentrations of lecithin and the negatively charged dye eosin B.

Eosin B	Hydrogel *D*_eff_ × 10^−10^ (m^2^·s^−1^)	Xerogel *D*_eff_ × 10^−10^ (m^2^·s^−1^)
R (0 wt.%)	2.3 ± 0.6	95 ± 13
0.5 wt.%	2.5 ± 0.5	12 ± 2·10^−2^
1 wt.%	2.5 ± 1.2	9 ± 3·10^−7^
2 wt.%	2.4 ± 1.3	4 ± 2

**Table 3:** Effective diffusion coefficients calculated from release dye experiments for physically crosslinked agarose hydrogels and xerogels with different concentrations of lecithin and the negatively charged amido black 10B.

Amido Black 10B	Hydrogel *D*_eff_ × 10^−10^ (m^2^·s^−1^)	Xerogel *D*_eff_ × 10^−10^ (m^2^·s^−1^)
R (0 wt.%)	2.6 ± 0.8	110 ± 53
0.5 wt.%	2.4 ± 1.0	18 ± 11
1 wt.%	2.2 ± 1.2	5.0 ± 0.4
2 wt.%	3.1 ± 0.1	6 ± 1

**Table 4:** Effective diffusion coefficients calculated from dye-release experiments for ionically crosslinked alginate hydrogels and xerogels with different concentrations of lecithin and the positively charged dye rhodamine 6G.

Rhodamine 6G	Hydrogel *D*_eff_ × 10^−10^ (m^2^·s^−1^)	Xerogel *D*_eff_ × 10^−10^ (m^2^·s^−1^)
R (0 wt.%)	2.9 ± 0.2	22 ± 2
0.5 wt.%	1.5 ± 0.1	—
1 wt.%	1.2 ± 0.1	—
2 wt.%	1.2 ± 0.1	—

**Table 5:** Effective diffusion coefficients calculated from dye-release experiments for ionically crosslinked alginate hydrogels and xerogels with different concentrations of lecithin and the negatively charged eosin B.

Eosin B	Hydrogel *D*_eff_ × 10^−10^ (m^2^·s^−1^)	Xerogel *D*_eff_ × 10^−10^ (m^2^·s^−1^)
R (0 wt.%)	2.0 ± 0.1	22 ± 8
0.5 wt.%	2.2 ± 0.2	0.4 ± 0.6
1 wt.%	2.2 ± 0.2	0.9 ± 1.2
2 wt.%	4.2 ± 0.3	1.7 ± 0.4

**Table 6:** Effective diffusion coefficients calculated from dye-release experiments for ionically crosslinked alginate hydrogels and xerogels with different concentrations of lecithin and the negatively charged amido black 10B.

Amido Black 10B	Hydrogel *D*_eff_ × 10^−10^ (m^2^·s^−1^)	Xerogel *D*_eff_ × 10^−10^ (m^2^·s^−1^)
R (0 wt.%)	1.5 ± 0.4	44 ± 2
0.5 wt.%	2.4 ± 0.4	1.8 ± 0.1
1 wt.%	1.7 ± 0.2	2.6 ± 0.1
2 wt.%	—	0.45 ± 0.02

**Table 7:** Effective diffusion coefficient (*D*_eff_) calculated from dye-release experiments for chemically crosslinked PVA-chitosan xerogels with different concentrations of lecithin and the positively charged dye rhodamine 6G.

Xerogel Sample (*c* of Lecithin)	R (0 wt.%)	0.5 wt.%	1 wt.%	2 wt.%
*D*_eff_ × 10^−10^ (m^2^·s^−1^)	9 ± 3	7 ± 1	1.9 ± 0.2	—

## Data Availability

The data used in this study are available on request from the corresponding author.

## Supplementary Materials

The following supporting information can be downloaded at: https://www.mdpi.com/article/10.3390/gels9050367/s1, Reference [36] is cited in the Supplementary Material. Figure S1: Influence of crosslinking on the release of rhodamine 6G; Figure S2: Preparation procedure of physically crosslinked agarose hydrogels; Figure S3: Average ATR-FTIR spectra of: (a) dried gel samples and (b) powder lecithin; Figure S4: Results of principal component analysis of the ATR-FTIR spectra of dried reference agarose hydrogel and agarose hydrogel with lecithin; Figure S5: Preparation procedure of ionically crosslinked alginate hydrogels; Figure S6: Results of principal component analysis of the ATR-FTIR spectra of dried reference alginate hydrogel and alginate hydrogel with lecithin, respectively; S7: Preparation procedure of chemically crosslinked PVA-chitosan hydrogels; Figure S8: Results of principal component analysis of the ATR-FTIR spectra of dried reference poly(vinyl alcohol)-chitosan hydrogel and poly(vinyl alcohol)-chitosan hydrogel with lecithin, respectively.
