# Supplementary material for: Lecithin as an Effective Modifier of the Transport Properties of Variously Crosslinked Hydrogels
Source: Gels. 2023 Apr 27;9(5):367. doi: 10.3390/gels9050367 (PMC10217736; doi:10.3390/gels9050367)
Supplement: Supplementary file 1 [file gels-09-00367-s001.zip › gels-2285541-Supplementary Materials.pdf]

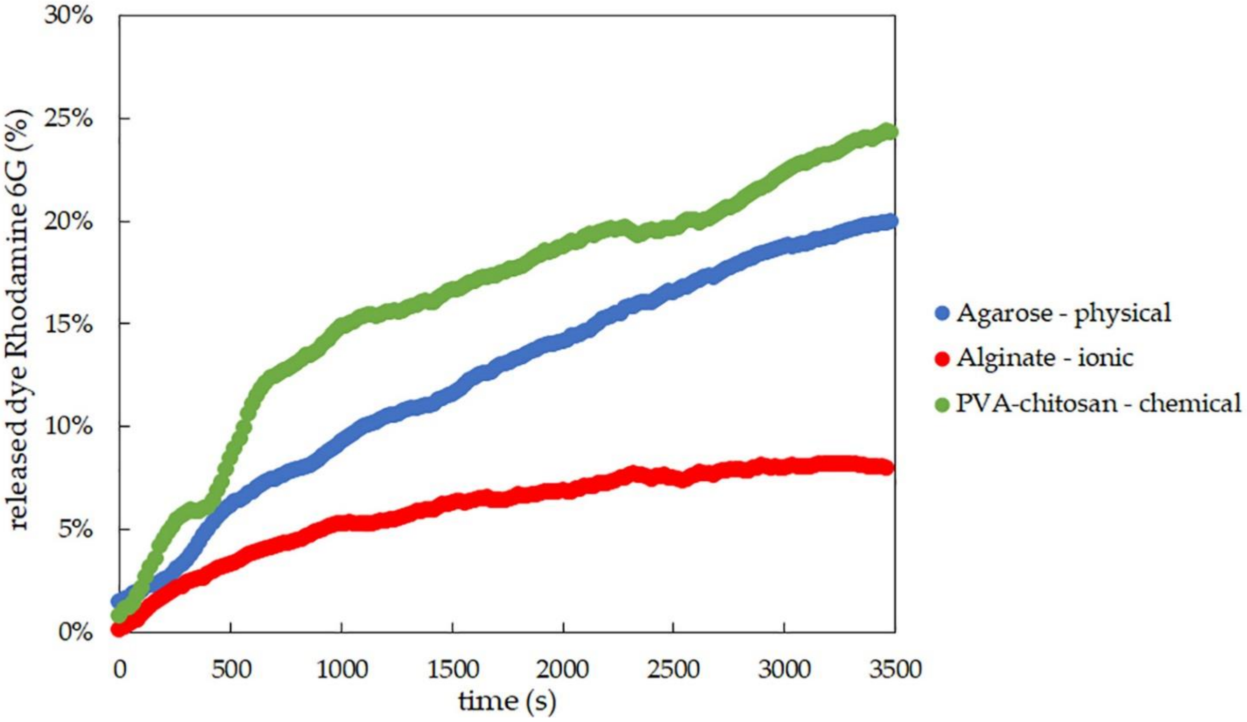

Figure S1 : Influence of crosslinking on the release of the Rhodamine 6G.

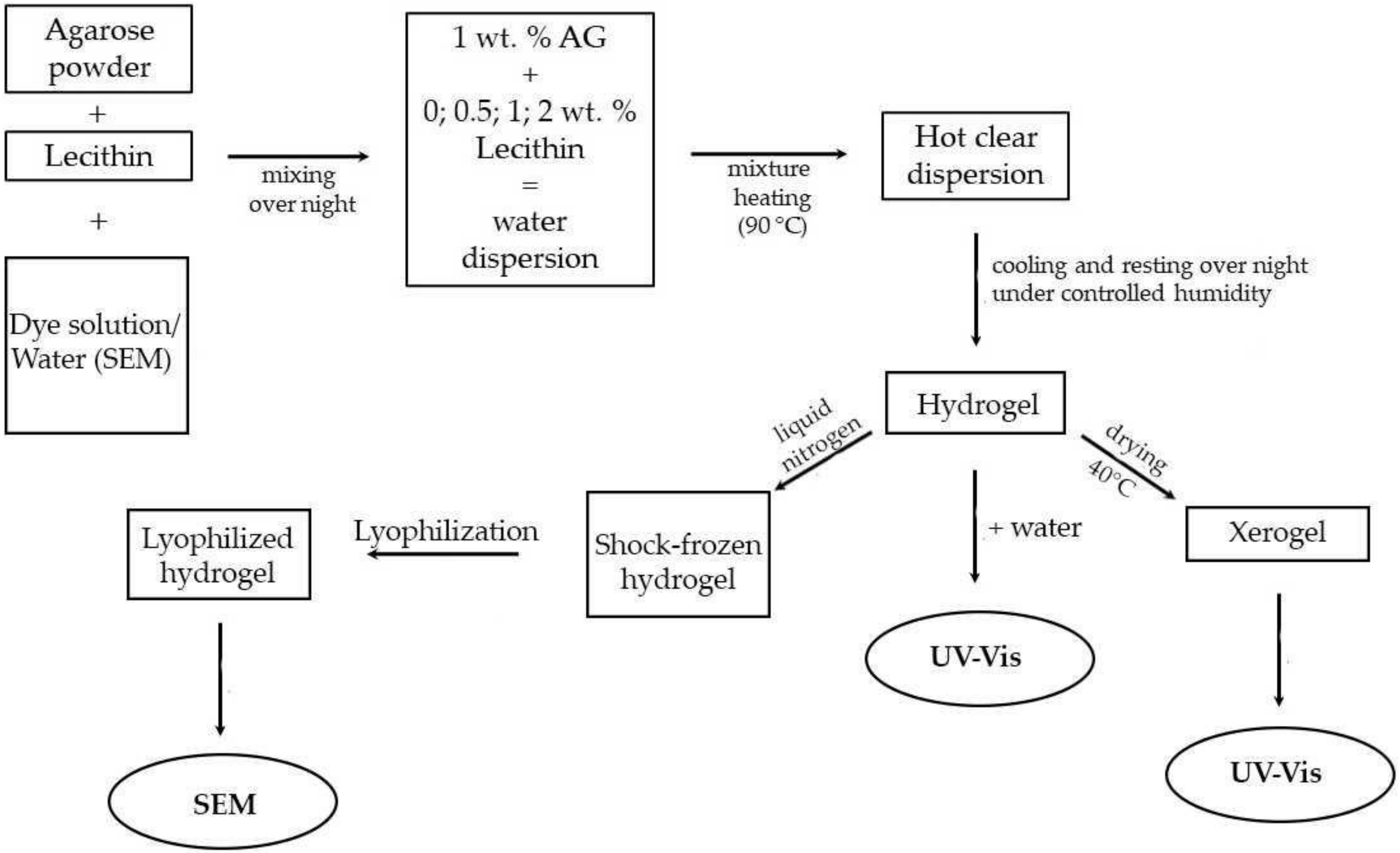

Figure S2: Preparation procedure of physically crosslinked agarose hydrogels.

Fourier transform infrared

Fourier transform infrared (FTIR) spectra of the samples were measured with iS50 FTIR spectrometer (Thermo Scientific, Waltham, MA, USA). Measurements were taken from a surface of a dried gels at ambient temperature (in an air-conditioned room) with the built-in single-reflection diamond attenuated total reflectance (ATR) crystal. Samples were analyzed in the standard mid-infrared spectral region (4000 – 400 cm^{-1}). An individual absorption spectrum was collected as an average of 32 scans with a resolution of 4 cm^{-1} (data spacing 0.5 cm–1). Each dried gel sample was analyzed at 10 randomly distributed spots (5 on each side) of its surface. FTIR spectra, shown in Figure S3a, represent an average of the ten spectra collected for an individual sample. Spectrum of the powder lecithin was measured in 5 repetitions, Figure S3b again shows the average spectrum., The whole-spectra PCA analysis was performed in the range of frequencies 4000 – 500 cm^{-1} using a standard multivariate principle component program written in-house using MATLAB software (MathWorks, Natick, MA, USA) at Institute of Scientific Instruments, Czech Academy of Sciences [36].

[36] Mlynáriková, K.; Samek, O.; Bernatová, S.; Růžička, F.; Ježek, J.; Hároniková, A.; Šiler, M.; Zemánek, P.; Holá, V. Influence of Culture Media on Microbial Fingerprints Using Raman Spectroscopy. *Sensors* **2015**, *15*, 29635-29647; doi:10.3390/s151129635.

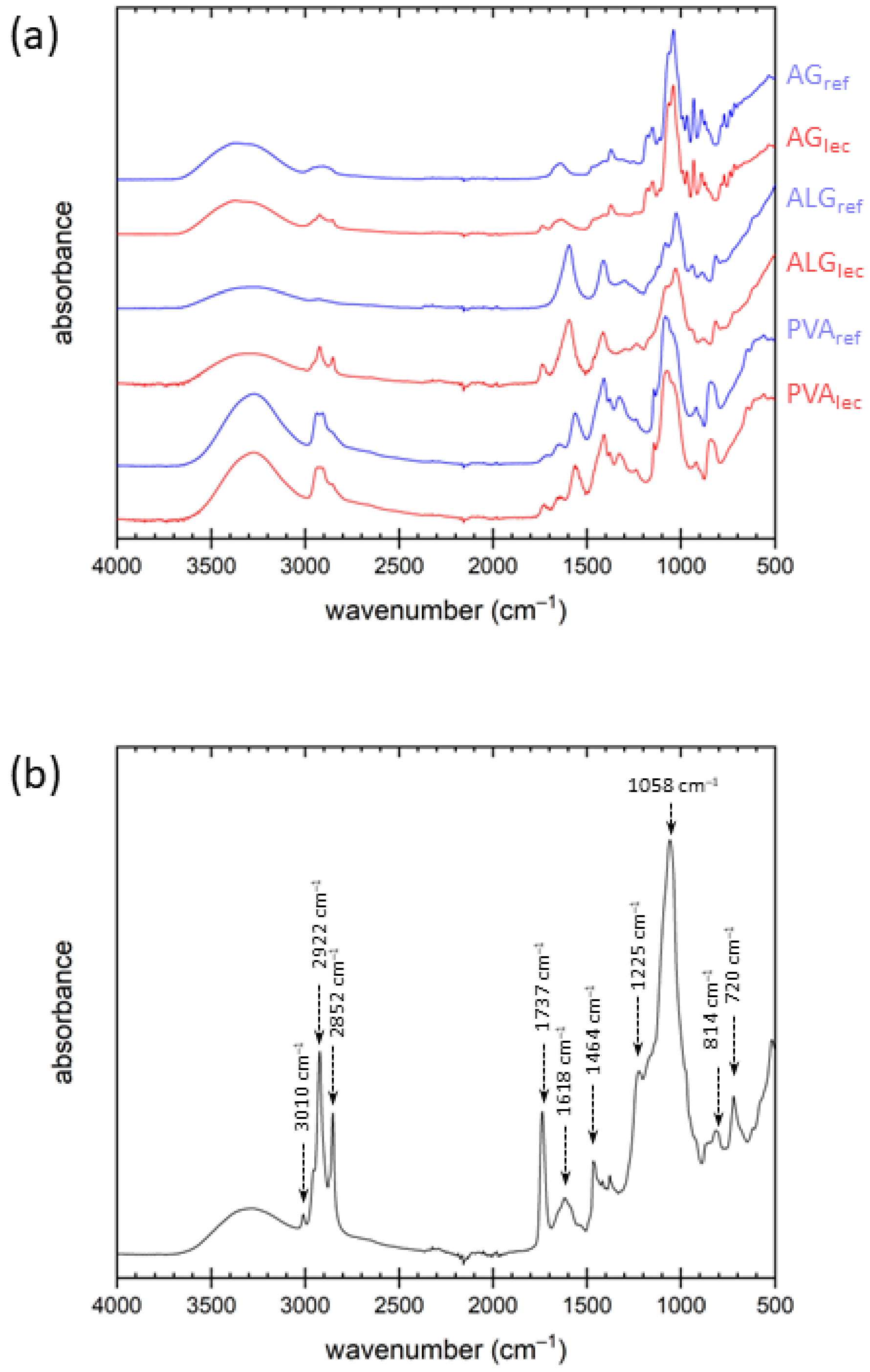

Figure S3: Average ATR-FTIR spectra of: (a) dried gel samples, (b) powder lecithin.

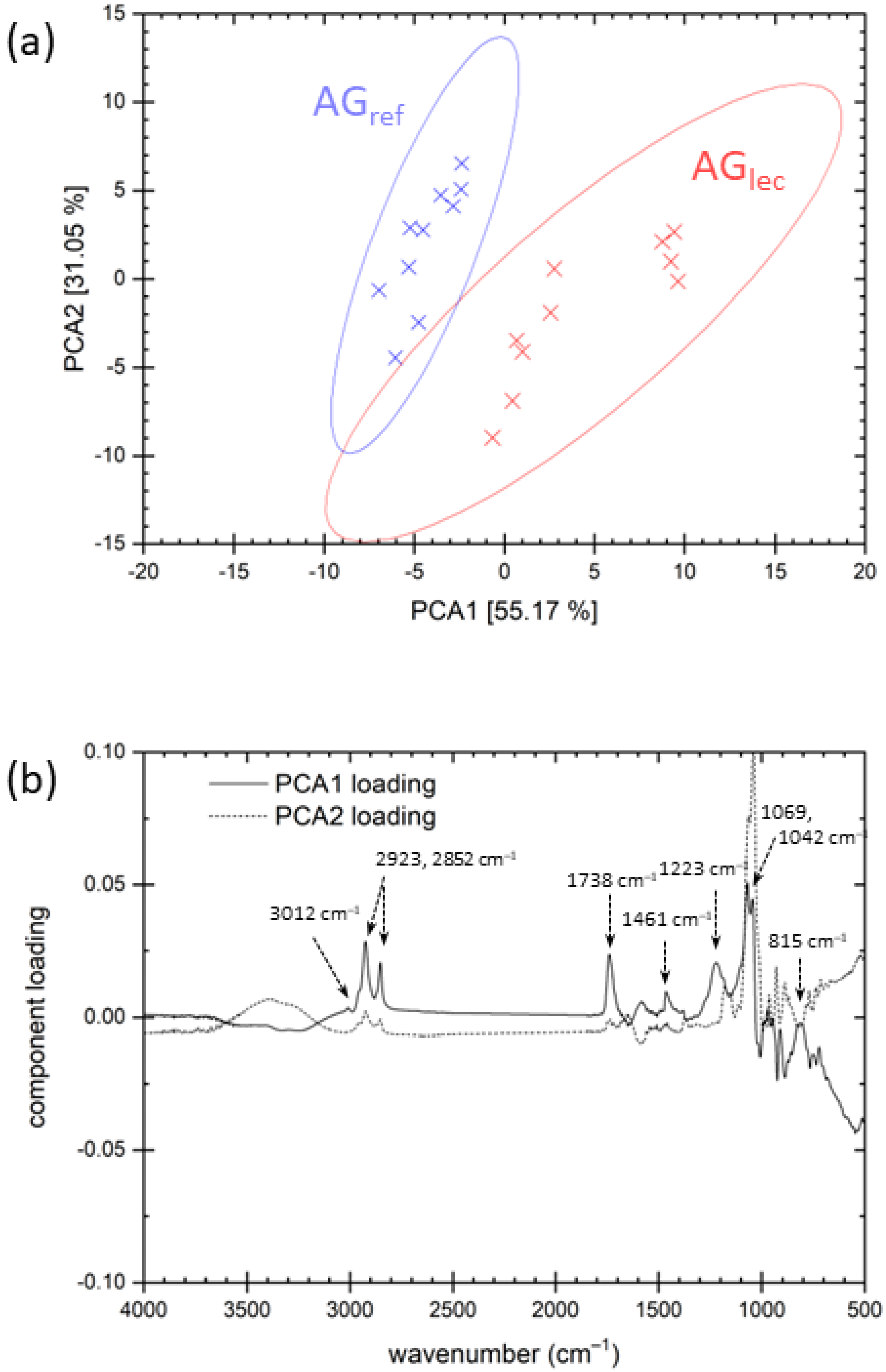

Figure S4: Results of Principal Component Analysis of the ATR-FTIR spectra of dried reference agarose hydrogel and agarose hydrogel with lecithin, respectively. (a) PCA biplot of the two major principal components. The value in the square bracket represents the relative variance that is composed in the respective principal component. Ellipses represent the 95% interval of confidence. (b) Loading of the two major principal components.

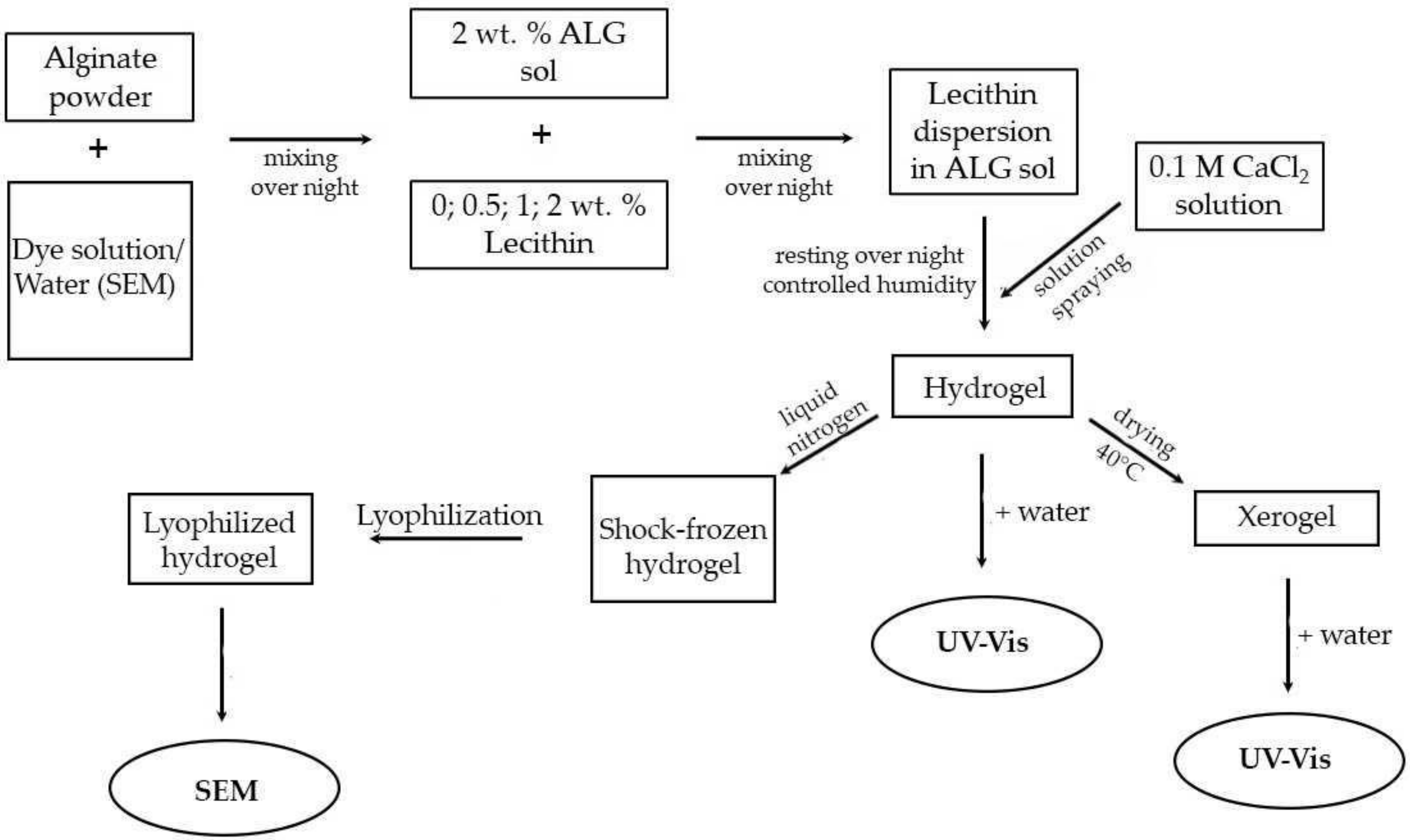

Figure S5: Preparation procedure of ionically crosslinked alginate hydrogels.

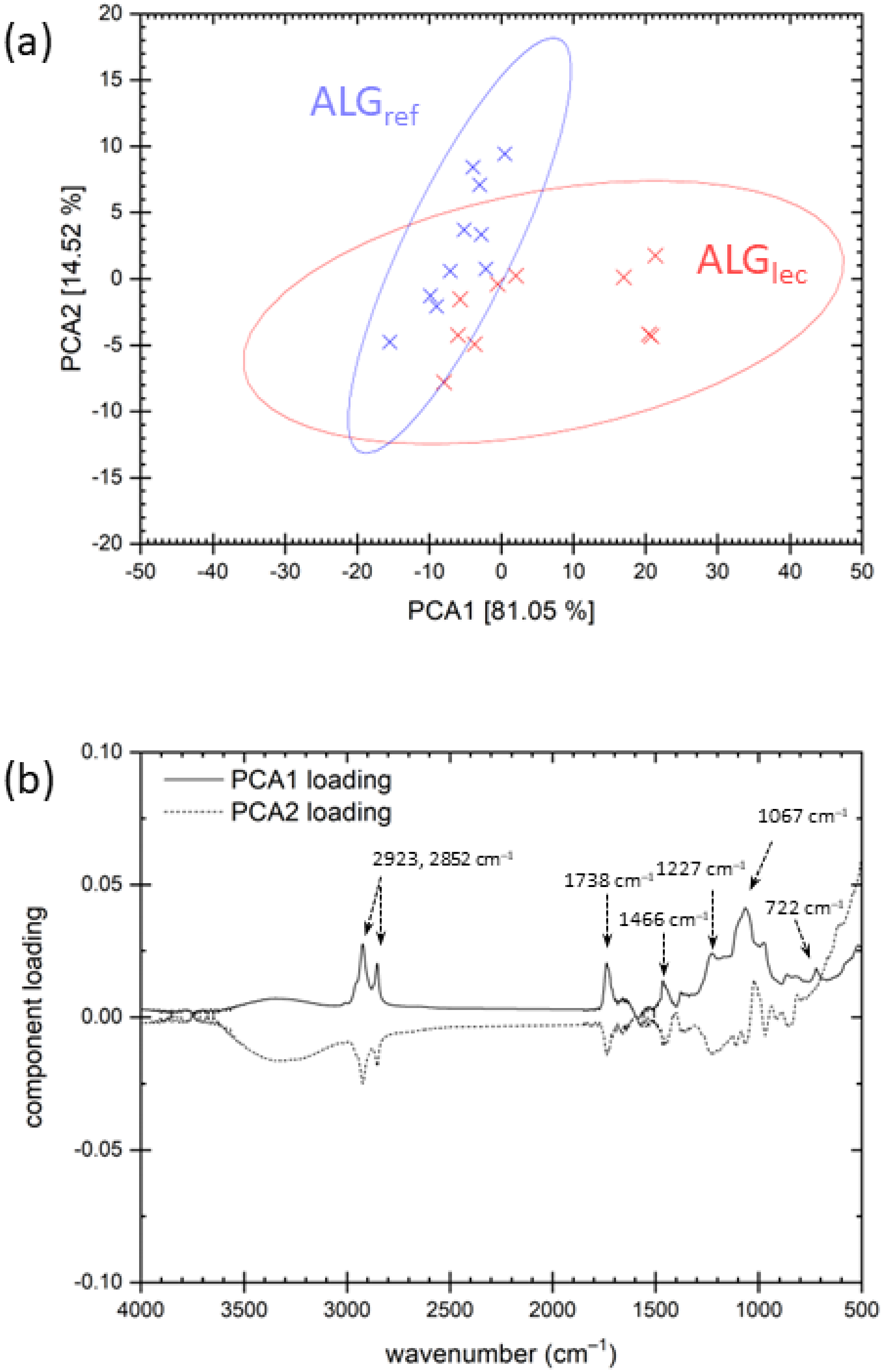

Figure S6: Results of Principal Component Analysis of the ATR-FTIR spectra of dried reference alginate hydrogel and alginate hydrogel with lecithin, respectively. (a) PCA biplot of the two major principal components. The value in the square bracket represents the relative variance that is composed in the respective principal component. Ellipses represent the 95% interval of confidence. (b) Loading of the two major principal components.

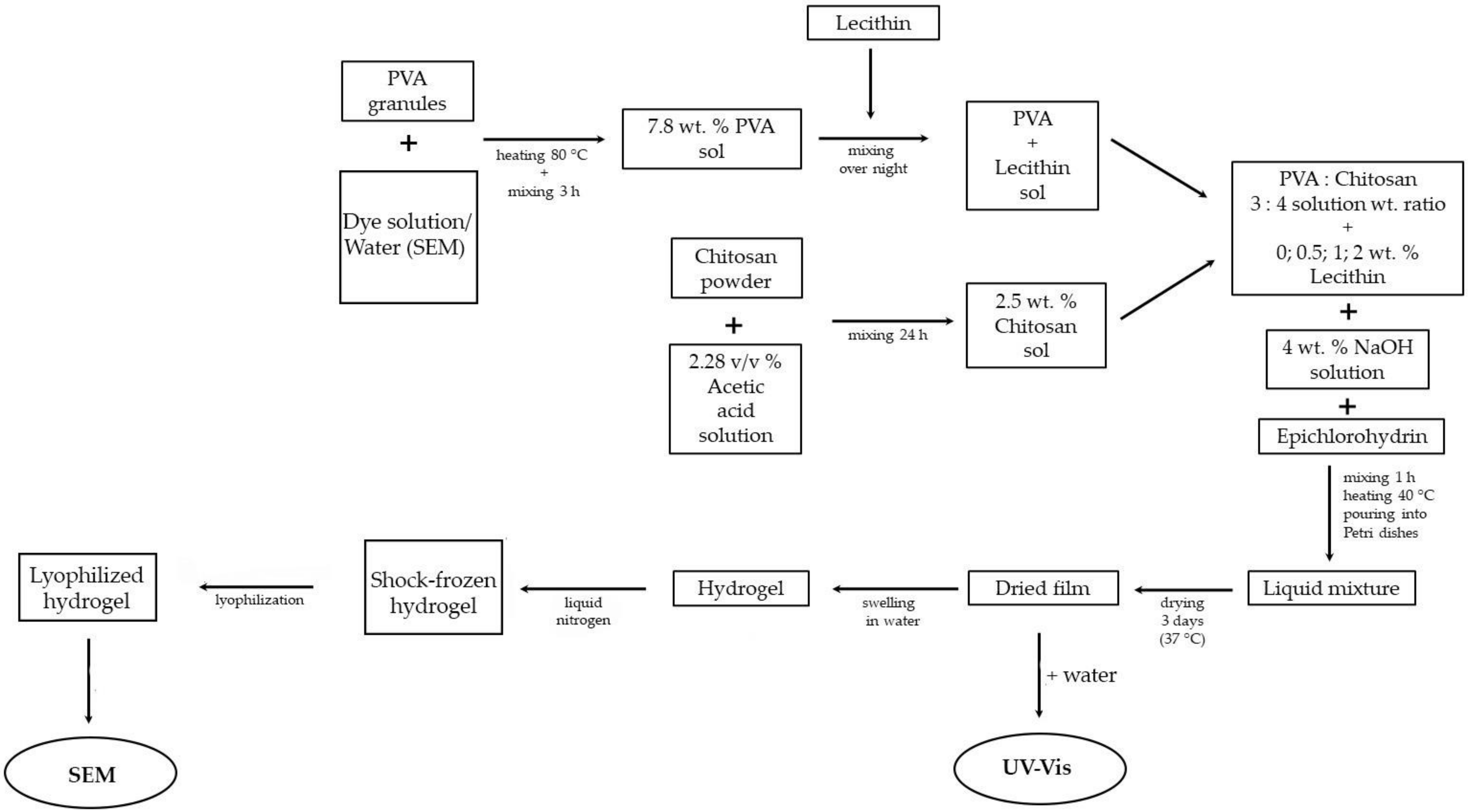

Figure S7: Preparation procedure of chemically crosslinked PVA-chitosan hydrogels.

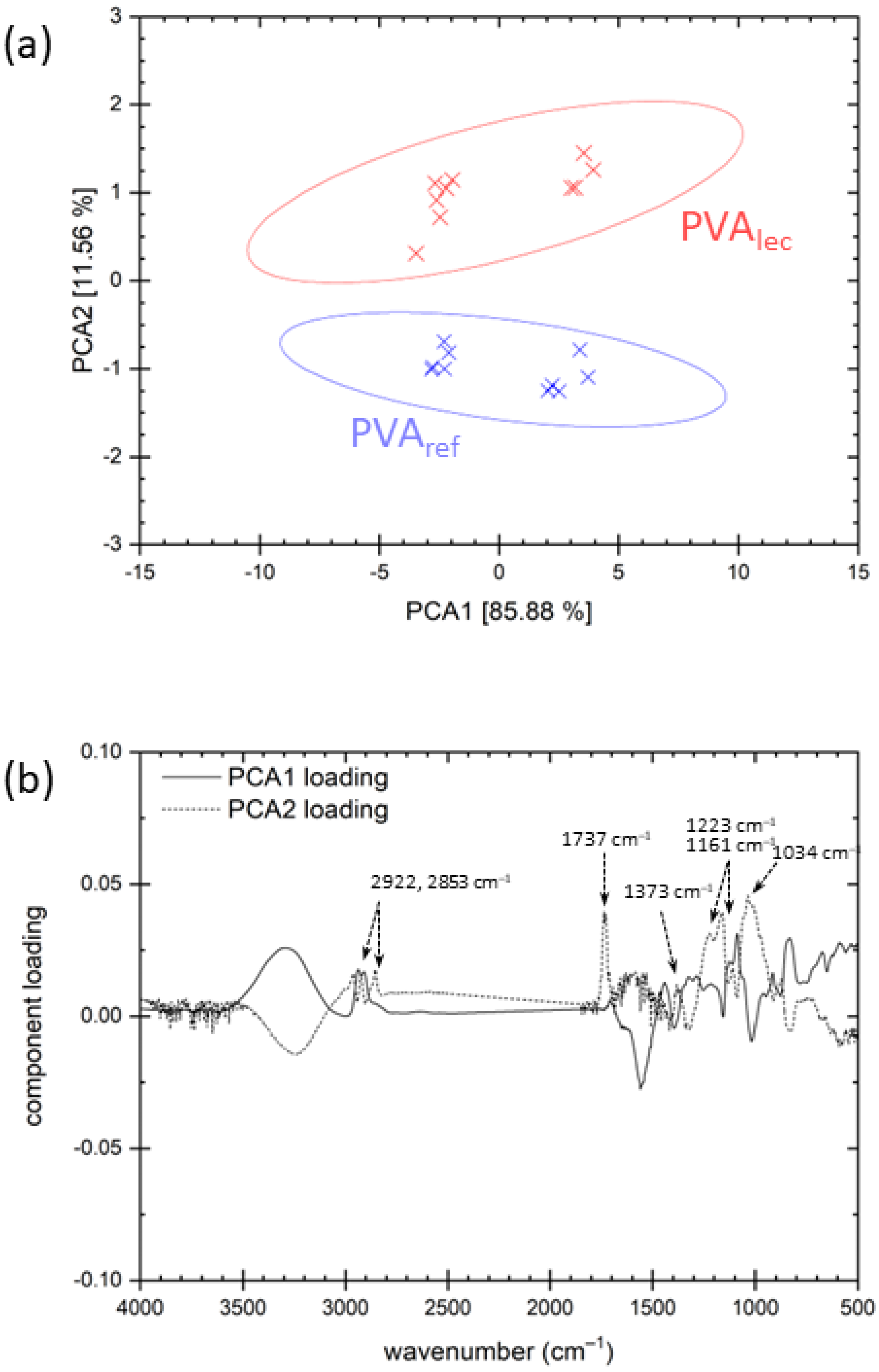

Figure S8: Results of Principal Component Analysis of the ATR-FTIR spectra of dried reference poly(vinyl alcohol)-chitosan hydrogel and poly(vinyl alcohol)-chitosan hydrogel with lecithin, respectively. (a) PCA biplot of the two major principal components. The value in the square bracket represents the relative variance that is composed in the respective principal component. Ellipses represent the 95% interval of confidence. (b) Loading of the two major principal components.
